# NRF2 Dysregulation and Therapeutic Insights Across Chronic Kidney Diseases

**DOI:** 10.3390/ijms26157471

**Published:** 2025-08-02

**Authors:** Tina Si Ting Lim, Kar Hui Ng, Yaochun Zhang

**Affiliations:** 1 Department of Paediatrics, Yong Loo Lin School of Medicine, National University of Singapore, Singapore 117549, Singapore; tinalst@nus.edu.sg (T.S.T.L.); paezyc@nus.edu.sg (Y.Z.); 2 Khoo Teck Puat-National University Children’s Medical Institute, National University Health System, Singapore 119074, Singapore

**Keywords:** NRF2, CKD, dysregulation, therapeutic, etiology

## Abstract

Chronic kidney disease (CKD) remains a global health burden, with limited therapeutic options that effectively target the underlying pathophysiology. Nuclear factor erythroid 2-related factor 2 (NRF2), a key regulator of oxidative stress and inflammation, has garnered significant attention as a potential therapeutic target in CKD. Despite encouraging preclinical results, no NRF2-targeted agents have achieved clinical approval for CKD treatment. This review synthesizes emerging evidence showing substantial heterogeneity in NRF2 activity across CKD subtypes, influenced by disease etiology, CKD stage, and rate of disease progression. We elucidate the key therapeutic implications across diverse CKD etiologies and highlight that the therapeutic efficacy of NRF2 activation depends on precise modulation tailored to disease context. Although NRF2 overactivation and the need for stage-dependent modulation are increasingly recognized, this review further delineates the consequences of indiscriminate NRF2 activation, demonstrating that its effects diverge across CKD etiologies and cellular contexts. These insights support a nuanced, context-specific approach to NRF2-targeted strategies and provide a framework to guide future drug development in CKD.

## 1. Introduction

The kidneys play a critical role in filtering both xenobiotic compounds and endogenous metabolic waste, rendering them particularly susceptible to oxidative stress and toxic exposure. As such, robust intrinsic defense mechanisms are essential to preserve renal function. One such mechanism is mediated by nuclear factor erythroid 2-related factor 2 (NRF2), a member of the cap’n’collar-basic leucine zipper family of DNA-binding transcription factors [1]. NRF2 expression is present throughout the kidney tissue, underlining its fundamental role in maintaining basal renal homeostasis. It functions as a redox-sensitive switch that turns off under homeostatic conditions and turns on during oxidative or electrophilic stress to activate cellular antioxidant and detoxification responses.

Chronic kidney disease (CKD) is characterized by the presence of kidney damage or a sustained reduction in estimated glomerular filtration rate (eGFR) below 60 mL/min/1.73 m^2^. It is associated with persistent oxidative stress and inflammation driven by upregulation of NADPH oxidases, mitochondria dysfunction, and reactive oxygen species (ROS) overproduction. This oxidative milieu contributes to cellular damage, tubular atrophy, and progressive interstitial fibrosis, eventually leading to loss of renal function [2,3,4]. Additionally, chronic inflammation in CKD is perpetuated by pro-inflammatory cytokines and immune cells, creating a vicious cycle that accelerates disease progression [5]. As a consequence, the NRF2 system is heavily implicated in the pathophysiology of CKD.

Recent studies have demonstrated that NRF2 expression and activity are highly heterogeneous across CKD and vary markedly with disease etiology, stage, and rate of progression. This molecular variability likely contributes to the inconsistent clinical outcomes observed with NRF2-modulating therapies [6]. In this context, the present review seeks to address a critical question: How do etiology-specific alterations in NRF2 signaling dictate the efficacy and therapeutic window of NRF2-targeted treatments across the spectrum of CKD? Additionally, we briefly explore therapeutic insights in renal fibrosis, a shared pathological feature underpinning diverse forms of CKD.

## 2. NRF2 Regulation

Beyond its canonical role in mitigating oxidative stress, NRF2 has been increasingly recognized as a key regulator of kidney physiology. For instance, NRF2 modulates salt and water homeostasis by regulating aquaporin-2 expression in the collecting ducts [7]. Furthermore, enhanced NRF2 activity has been shown to increase glomerular filtration rate and modulate glomerular hemodynamics by regulating sodium–glucose cotransporter 2 (SGLT2) expression in proximal tubules [8,9]. These findings suggest that maintaining an appropriate level of NRF2 activity is paramount to physiological homeostasis. Indeed, NRF2 expression is strongly regulated by various mechanisms described in this section.

### 2.1. Transcriptional, Epigenetic, and Post-Transcriptional Regulation

NRF2 expression is modulated at the transcriptional level by a combination of promoter elements and upstream signaling pathways [10]. The *NFE2L2* promoter encoding NRF2 contains a xenobiotic response element (XRE) which serves as a binding site for transcription factor aryl hydrocarbon receptor (AHR) [11]. AHR mediates NRF2 induction in response to uremic toxins which accumulate in CKD. The *NFE2L2* promoter also contains a NF-kB binding site [12]. NF-kB is known to be activated in response to oxidative signals, and upon binding to the *NFE2L2* promoter, can enhance NRF2 transcription. Notably, NRF2 itself may regulate its own expression through the binding of AREs on its own promoter, creating a positive feedback loop under oxidative stress conditions [13].

*NFE2LE* also undergoes strong epigenetic and post-transcriptional regulation [14]. DNA methylation of CpG islands in the *NFE2L2* promoter has been associated with NRF2 silencing. This was seen in prostate tumors [15] and aged or fibrotic kidneys where increased expression of DNA methyltransferases is observed [16]. Similarly, KEAP1 promoter hypomethylation has been linked to elevated KEAP1 expression, in turn leading to NRF2 degradation [17,18]. Histone acetylation of NRF2 by CBP/p300 has been associated with increased promoter-specific DNA binding by NRF2 [19], while histone deacetylases repress NRF2 expression and have been shown to be elevated in some models of CKD [20,21]. Additionally, numerous microRNAs (miRNAs) can regulate NRF2 expression. One prominent example is miR-144-3p, which was markedly upregulated in models of diabetic and hypertensive nephropathy. miR-144-3p was shown to bind directly to the 3′ UTR of *NFE2L2* mRNA, suppressing NRF2 expression [22,23]. Long non-coding RNAs can also impact NRF2 expression by sequestering miRNAs that would otherwise bind and degrade *NFE2L2* mRNA (e.g., TUG1) [23].

### 2.2. KEAP1-Dependent Regulation

**Figure 1:**
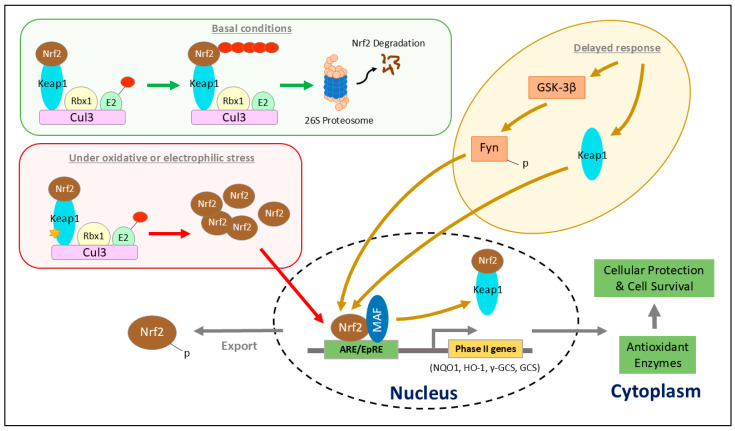
Oxidative/electrophilic stress and NRF2/KEAP1 signaling pathway. Under basal conditions (green box), Kelch-like ECH-associated protein 1 (KEAP1) serves as an adaptor to link nuclear factor erythroid 2-related factor 2 (NRF2) to the ubiquitin ligase cullin3-ring box 1 (Cul3-Rbx1) complex and leads to proteasomal degradation of NRF2. Upon exposure to reactive oxygen species (ROS) or electrophiles (red box), key cysteine residues within KEAP1 undergo covalent modification, impairing its ability to repress NRF2. This disruption permits NRF2 accumulation, followed by its translocation into the nucleus, where it heterodimerizes with small musculo-aponeutoric fibrosarcoma (sMAF) and binds to the antioxidant response element (ARE) to initiate the transcription of cytoprotective phase II detoxifying enzymes (NADPH-dehydrogenase quinone 1 (NQO1), heme oxygenase-1 (HO-1), glutamylcysteine synthetase (GCS)). As a delayed response to oxidative/electrophilic stress (yellow oval), glycogen synthase kinase-3β (GSK-3β) is activated and phosphorylates Fyn kinase. Following this, nuclear translocation of phosphorylated Fyn and KEAP1 phosphorylates NRF2 at Tyr568, which results in NRF2 nuclear exporting and degradation. Additionally, NRF2 degradation is facilitated by prothymosin α-mediated nuclear translocation of INRF2. Together, these cytoplasmic and nuclear degradation mechanisms tightly regulate NRF2 levels, ensuring rapid termination of its transcriptional activity once homeostasis is restored.

Under basal conditions, Kelch-like ECH-associated protein 1 (KEAP1) phosphorylates NRF2. This allows the bridge formation between NRF2 and E3 ubiquitin ligase constituted by the cullin3-ring box 1 (Cul3-Rbx1) [24,25,26]. NRF2 is then targeted for proteasomal degradation by ubiquitination. Under oxidative or electrophilic stress, ROS modify thiol side chains of critical cysteine residues on KEAP1, causing conformational changes that include intramolecular disulfide bridges. This triggers the release of NRF2 and the subsequent proteasomal degradation of KEAP1 [27,28,29]. Accumulated NRF2 translocates to the nucleus, where it forms a heterodimer with small musculo-aponeutoric fibrosarcoma (sMAF) transcription factors [30,31]. sMAF-NRF2 recognizes and binds to the 5′-RTGACNNNGC-3′ sequence, known as antioxidant response elements (AREs) in target gene promoters [32], leading to the upregulation of a broad cytoprotective gene sets including detoxification enzymes (e.g., NADPH-dehydrogenase quinone 1 (NQO1)) and antioxidant enzymes (e.g., Heme oxygenase-1 (HO-1), glutathione S-transferases (GSTs)) [33,34]. These enzymes play a role in mitigating oxidative stress, detoxification pathways and glutathione synthesis, highlighting NRF2’s role in regulating kidney homeostasis (Figure 1).

### 2.3. KEAP1-Independent Regulation

In addition to KEAP1-mediated regulation, the NRF2 pathway is modulated by multiple kinase signaling pathways. For example, phosphorylation of NRF2 by glycogen synthase kinase-3β (GSK-3β) promotes its b-TrCP-mediated nuclear export and proteasomal degradation [35,36]. Conversely, AMP-activated protein kinases (AMPKs) and mitogen-activated protein kinases (MAPKs) positively regulate NRF2 activation, linking NRF2 signaling to cellular metabolic status [37,38,39]. Several other proteins have also been identified by previous studies to play a role in NRF2 regulation. Namely, CR6-interacting factor 1 (CRIF1) promotes NRF2 ubiquitination and its subsequent proteosomal degradation by interaction with both its N- and C-terminal regions [40]. Additionally, the seven in absentia homolog 2 (Siah2) protein suppresses NRF2 through a binding motif [41].

## 3. Mechanisms of NRF2 Dysregulation in Chronic Kidney Diseases

Oxidative stress, inflammation, metabolic dysfunction and fibrosis are hallmarks of CKD, all of which converge on the cytoprotective KEAP1-NRF2 signaling axis. Understanding the mechanisms by which NRF2 signaling becomes dysregulated, either through aberrant activation or suppression, is critical for designing targeted therapeutic strategies. The following sections cover the molecular pathways through which NRF2 influences CKD pathogenesis.

### 3.1. Redox Balance and Antioxidant Defense

Systemic and renal oxidative stress is typically present in patients with CKD, caused by increased ROS production and impaired antioxidant capacity. In response to oxidative stress, NRF2 regulates key antioxidant defenses that maintain redox balance. These defenses include glutathione (GSH) synthesis, antioxidant enzymes (e.g., thioredoxin, GSH peroxidase, etc.), and detoxification enzymes (e.g., NQO1) [34,42]. Collectively, these NRF2 target proteins work in concert to neutralize superoxide, peroxides, and electrophiles. This is achieved by directly scavenging free radicals, regulating downstream targets or detoxifying reactive chemicals [43]. These mechanisms ensure that physiological ROS production does not escalate into oxidative stress.

Dysregulation of the NRF2 antioxidant defense has been well documented in CKD, with experimental studies supporting its functional importance. In animal models of induced kidney damage, NRF2 deficiency consistently exacerbates oxidative injury—unilateral ureteral obstruction (UUO) in *Nrf2*-knockout mice leads to significantly increased lipid peroxidation and tissue damage correlating to loss of antioxidant response [44]. Treatment with sulforaphane, a known NRF2 activator, reduces ROS levels and DNA oxidation in kidney cells exposed to uremic toxins. Sulforaphane also restored antioxidant enzyme expression, protecting glomeruli from oxidative injury in models of diabetic nephropathy [45,46].

Clinical evidence implicates oxidative stress as a key modulator of the NRF2 pathway in CKD. Biopsy samples from patients with CKD stages 2–3 have demonstrated increased renal expression of NRF2 and its downstream targets. This suggests a compensatory elevation of NRF2 activity in response to moderate oxidative stress in early CKD [47]. Another study, in which oxidative stress was transiently induced using tin–protoporphyrin administration, showed significant increases in plasma concentrations of NQO1, HO-1, and ferritin, all of which are regulated by NRF2 [48]. However, it should be noted that these proteins can also be regulated through NRF2-independent pathways. As such, increased expression alone does not unequivocally indicate NRF2 activation.

### 3.2. Inflammation

Oxidative stress and inflammation are interrelated processes that jointly contribute to the progression of CKD. The upregulation of inflammatory biomarkers in CKD has been well documented [49,50]. NRF2 is widely recognized for its anti-inflammatory properties, which are thought to be mediated through three principal mechanisms. Firstly, NRF2 suppresses inflammation indirectly by reducing ROS, which are key activators of pro-inflammatory signaling pathways. Secondly, NRF2 directly competes with transcriptional regulator NF-κB, a master driver of the inflammatory response, with the CREB-binding protein (CBP/p300) as a co-activator [51]. More specifically, when NRF2 is active, CBP is sequestered, dampening NF-κB-mediated cytokine release. Thirdly, NRF2 target genes regulate the NF-κB pathway—HO-1 induction, for example, has been shown to reduce renal inflammatory cell infiltration. Mechanistically, HO-1 byproducts can inhibit the phosphorylation of IκB and the p65 subunit of NF-κB, preventing NF-κB activation [52]. Sulforaphane, a potent activator of NRF2, was found to block IκBα degradation in kidney cells, thereby retaining NF-κB in the cytosol and blunting the inflammatory cascade [53].

Experimental evidence supports the association between inflammation in CKD and dysregulation of the NRF2 system. In a 5/6 nephrectomy model, inactivation of NRF2 resulted in renal inflammation and increased NF-κB activity, leading to monocyte chemoattractant protein-1 (MCP-1) and cyclooxygenase-2 (COX-2) upregulation [54]. Similarly, CKD rat models exhibited elevated expression of pro-inflammatory molecules, including COX-2 and MCP-1, correlating with decreased levels of NRF2 and its target gene products, NQO1, catalase, and copper zinc superoxide dismutase (CuZn-SOD) [55]. Furthermore, in a mice model with Keap1 hypomorphism, enhanced NRF2 activation was associated with reduced renal expression of MCP-1 and interleukins, suggesting that NRF2 attenuates angiotensin II-induced ROS production and ameliorates inflammation [56].

### 3.3. Fibrosis

Renal fibrosis is a hallmark of CKD progression, contributing to progressive nephron loss through accumulation of extracellular matrix proteins, tubular atrophy, and glomerulosclerosis [57,58]. Persistent oxidative stress and inflammation are key drivers of fibrosis [59]. Through its antioxidant and anti-inflammatory roles, NRF2 activation suppresses the stimuli that enhances profibrotic signaling, indirectly playing a critical role in the defense against fibrosis. Importantly, NRF2 also works to directly repress profibrotic gene expression by inhibiting the TGF-β/SMAD and PI3K/Akt signaling pathways [60,61]. A study by Oh et al. showed that NRF2 inhibited TGF-β/SMAD3 signaling independent of ARE signaling, suggesting direct physical interaction between NRF2 and SMAD3 [62]. NRF2 activation also upregulates SMAD7, which competes with SMAD3 and disrupts pro-fibrotic signaling [63]. Pharmacological induction of NRF2 in renal proximal tubules has been shown to mitigate ischemia–reperfusion tubular necrosis and subsequent interstitial fibrosis by upregulating cytoprotective genes and repressing profibrotic gene expression [64]. Another study conducted by Qin et al. demonstrated that sinomenine exerted anti-fibrotic effects in an NRF2-dependent manner, primarily by inhibiting the TGF-β/SMAD and WNT/β-catenin pro-fibrotic signaling pathways [65]. These studies provide further evidence of NRF2’s protective role in fibrosis.

Experimental models have elucidated the role of NRF2 dysregulation in CKD. Given the role that oxidative stress and inflammation plays in exacerbating fibrosis, it can be inferred that the protective role of NRF2 in fibrosis is dependent on disease progression; while short term UUO in mice results in NRF2-dependent antioxidant mechanisms, longer term obstruction results in decreased antioxidative response, increased oxidative stress, inflammation, and eventually fibrosis. This was shown in UUO mice models, where *Nrf2*-knockout mice saw an increase in fibrotic (TGF-β1, fibronectin, a-SMA) and inflammatory markers (TGF-β, TNF, IL-6, IL-1b, F4/80) compared to control mice, and antioxidative responses were decreased in *Nrf2*-knockout mice at day 14 [44]. Using rat models, Sun et al. showed that hippuric acid (HA), a type of protein-bound uremic toxin (PBUT) that accumulates in CKD, disrupted the NRF2-driven antioxidant response, leading to disrupted antioxidant networks and ROS accumulation, resulting in renal fibrosis [66].

### 3.4. Metabolic Signaling

In the kidneys, regulation of energy production and lipid homeostasis maintains metabolic balance. Dysregulation of these processes can lead to mitochondrial dysfunction, lipid toxicity, and a pro-inflammatory microenvironment, contributing to kidney disease [67,68]. For example, increased fatty acid oxidation and lipid accumulation has been shown to contribute to the pathogenesis of diabetic kidney disease [9,68]. NRF2 plays an essential role in regulating these functions by modulating genes involved in lipid and carbohydrate metabolic pathways. For instance, activation of NRF2 inhibits fatty acid synthesis by suppressing FASN and SCD1 [69,70]. At the same time, it appears to stimulate fatty acid oxidation, although exact mechanisms remains unclear [71]. NRF2 inhibition has also been shown to suppress the expression of ACSL1 indirectly through oxidative stress, leading to increased lipid deposition [72]. Additionally, NRF2 activation upregulates the pentose phosphate pathway by redirecting glucose metabolism [73,74,75].

Metabolic reprogramming is evidenced in CKD models, both in vitro and in vivo. In patients with type 2 diabetes and CKD, decreased NRF2 expression correlated with hyperglycemia and elevated triglycerides [76]. NRF2 silencing has been implicated in mitochondrial injury and NLRP3 inflammasome activation in vivo and in vitro, contributing to hyperlipidemia-induced renal injury [77]. In diabetic nephropathy mice models, *Nrf2* knockout attenuates DKD progression through the downregulation of its target genes including angiotensinogen, *Sglt2*, scavenger receptor CD36, and fatty-acid-binding protein 4 [9]. Experimentally, hyperglycemia suppressed NRF2 and its downstream gene target in HK2 proximal tubular cells, impairing oxidative phosphorylation [78]. In autosomal polycystic kidney disease (ADPKD) models, metabolic reprogramming promotes a shift towards the pentose phosphate pathway (PPP) and fatty acid biosynthesis, and a decrease in fatty acid oxidation and oxidative phosphorylation [79,80]. Another study investigating lipid metabolism in patients with CKD stages 3–5 showed association between abnormal lipid metabolism with PPARy, TRP channels, and RAS signaling [81]. While these studies did not directly implicate NRF2 involvement, the cellular programs and signaling pathways involved are typically regulated by NRF2. This permits the inference that NRF2 signaling may contribute, even if indirectly, to metabolic dysregulation in CKD. These findings underscore the need for further investigation into the role of NRF2 in metabolic reprogramming during CKD progression.

### 3.5. NRF2 Dysregulation in CKD Stages

Overall, NRF2 is typically regarded as a protective transcription factor activated in response to oxidative stress. The effects of NRF2 outlined in Section 3.1, Section 3.2, Section 3.3 and Section 3.4 collectively support a causal role of NRF2 downregulation in CKD progression. Yet, studies have indicated that NRF2 signaling follows a biphasic, stage dependent pattern [47]. Transient activation of NRF2 is typically protective in early CKD, but when oxidative and inflammatory stress persist, continued or excessive activation can paradoxically intensify renal injury.

In early or mild CKD, there is evidence of compensatory NRF2 activation and upregulation of antioxidant and cytoprotective genes. However, advanced CKD stages are frequently associated with repression of the NRF2 system even as oxidative stress and inflammation intensifies. Indeed, in a systematic review of 32 CKD studies, NRF2 was found to be decreased in a majority of the studies, with NQO1 levels particularly reduced in advanced stages of CKD. These findings highlight a progressive impairment of the NRF2-mediated antioxidant defense system as the disease advances [82].

Patients with advanced CKD exhibiting the highest levels of inflammatory cytokines and uremic toxins typically demonstrate the lowest NRF2 activity. In ADPKD, kidneys with a greater cystic burden show significantly reduced NRF2 expression [83]. In diabetic kidney disease, NRF2 is initially upregulated during early diabetes, whereas advanced diabetic nephropathy is characterized by diminished NRF2 levels [84]. A comparable stage-dependent pattern of NRF2 dysregulation has been observed in lupus nephritis (LN), where glomerular NRF2 and NQO1 expression increases in early-stage LN but declines as the disease progresses [85]. Collectively, these findings imply that the NRF2 pathway is initially activated but subsequently suppressed as disease burden overwhelms cellular defense mechanisms.

Given the complexity of NRF2 dysregulation, understanding the specific mechanisms and contexts in which NRF2 is repressed has direct implications for therapy. The progressive nature of CKD means that sustained activation of NRF2 is not universally beneficial. Rush et al. found that NRF2 activation exacerbated proteinuria in CKD mice models and was upregulated in patients with focal segmental glomerulosclerosis (FSGS) and diabetic nephropathy [86]. Additionally, another study showed that overexpression in renal proximal tubular cells can exacerbate blood glucose and kidney injury in diabetes [8]. Similar results have also been seen in an AS mice model [87]. This shows that activation of NRF2 must be carefully calibrated, and suggests that etiological differences also inform context-dependent therapeutic methods. Specifically, NRF2 activity is neither uniformly suppressed nor enhanced across all CKD subtypes [6,82]. Hence, it will be prudent to take into account disease etiology when designing therapeutic modulation of NRF2.

## 4. Therapeutic Insights Across CKD Etiologies

To date, the beneficial effects of NRF2 activation on renal function have been demonstrated in numerous experimental studies. Given its cytoprotective role, NRF2 represents a promising therapeutic target for kidney diseases. Its pharmacological induction is also currently being evaluated in various clinical trials. The most notable results thus far have been observed in studies targeting diabetic nephropathy. In this section, we explore therapeutic insights from several forms of CKD in which the role of NRF2 has been investigated. This section will highlight how etiology-specific abnormalities in NRF2 signaling may shape targeted therapeutic strategies.

### 4.1. Diabetic Kidney Disease (DKD)

In DKD, high glucose and lipid levels cause increased oxidative stress, chronic inflammation, and disruption of oxidative phosphorylation in kidney cells [67]. Metabolic reprogramming is a key mechanism contributing to disease pathogenesis, and key signaling pathways involved an overlap with NRF2 regulation. In patients with type 2 diabetes, those who develop DKD show significantly lower NRF2 mRNA expression than diabetic patients without DKD [88]. Early DKD shows activated NRF2 as a compensatory antioxidant response, demonstrated by elevated NRF2 levels and unchanged KEAP1 levels in diabetic patients with CKD stages 0–3b [8]. However, advanced DKD was associated with a reduction in NRF2 protein, accompanied by upregulation of KEAP1, suggesting increased degradation of NRF2 in advanced DKD [89]. In another study, a decrease in NRF2 levels was found in DKD patients compared to healthy kidney tissue [78].

NRF2 has attracted interest as a therapeutic target in DKD. Numerous compounds that modulate NRF2 activity through diverse mechanisms have been tested for their potential efficacy in DKD management. Amongst them, epigallocatechin-3-gallate (EGCG) has shown potential in preventing disease progression when administered at an early stage by modulating NRF2 and its negative regulators [90,91]. In brief, some compounds work by inhibiting ferroptosis, which has been shown to delay progression of DKD [92], while others inhibit the NLPR3 inflammasome or modulate various NRF2 pathways. These compounds and their mechanisms are summarized in Table 1. However, broad NRF2 activation may have unintended effects—for instance, recent data suggests that NRF2 activation in diabetic kidneys can upregulate genes like angiotensinogen (*AGT*) and *SGLT2*. This potentially exacerbates hypertension and hyperglycemic stress in the kidney [8,9]. Furthermore, pharmacological tests have previously yielded conflicting results. The most notable example is bardoxolone methyl, a potent NRF2 activator. In a phase 2 trial (BEAM; NCT00811889), bardoxolone methyl significantly increased eGFR over 52 weeks [93]. However, the subsequent phase 3 BEACON trial (NCT01351675) was terminated prematurely due to an increased incidence of heart failure and mortality among treated patients, despite improvements in eGFR [94]. Both studies raised concerns regarding the exacerbation of albuminuria. Thus, although NRF2-activating compounds have shown renoprotective promise, their clinical application in DKD warrants cautious and rigorous evaluation.

The risk of adverse effects emphasizes that the diabetic context demands a balanced approach to NRF2 modulation. In addition to evaluating long-term safety and potential off-target effects, careful consideration must be given to achieving an optimal balance between insufficient and excessive NRF2 activation. Future therapeutic strategies may benefit from combination treatments to mitigate downstream adverse effects of NRF2 activators. For instance, co-administration of L-ergothioneine and metformin has been shown to attenuate renal dysfunction while simultaneously reducing hyperglycemia in type 2 diabetic rat models [95]. Similarly, the combination of metformin and salvianolic acid enabled dual regulation via metformin-mediated activation of AMP-activated protein kinase (AMPK) and salvianolic acid-mediated NRF2 signaling [96]. Crucially, as illustrated in the case of bardoxolone methyl, increases in eGFR alone are insufficient to establish therapeutic efficacy. Future clinical trials should incorporate more comprehensive endpoints to accurately assess both the efficacy and safety of NRF2-targeted therapies.

**Table 1:** NRF2 modulators tested in DKD and their mechanisms.

NRF2 Modulators	Chemical Class	Study Design	Dosage and Duration of Treatment	Pathway/ Mechanism	Reference
**Lixisenatide**	Polypeptide	In vivo (STZ-induced diabetic rats)	10 µg/kg for 4 weeks	Modulate NRF2/HO-1 signaling pathway	[97]
Telmisartan	Benzimidazole	In vivo (STZ-induced diabetic rats)	5 or 10 mg/kg for 8 weeks		[98]
Digitoflavone	Flavonoid	In vitro (SV40-MES13 cells) In vivo (STZ-induced diabetic mice)	25 or 50 mg/kg		[99]
Notoginsenoside R1	Saponin	In vitro (HK-2 cells) In vivo (db/db mice)	30 mg/kg/day for 20 weeks		[100]
Sinapic acid	Cinnamate	In vivo (STZ-induced diabetic rats)	20 or 40 mg/kg for 8 weeks		[101]
Compound centella	Herbal and Dietary Supplements	In vivo (STZ-induced diabetic rats)	0.8 g Centella asiatica, 0.8 g Astragalus membranaceus, and 0.4 g Tripterygium wildorfii per rat for 112 days		[102]
Phosphocreatine	Amidine	In vitro (NRK-52E cells) In vivo (STZ-induced diabetic rats)	In vitro: 10, 20, or 40 mm for 4 h In vivo: 25 or 50 mg/kg for 72 h		[103]
Artemisinin	Sesquiterpene	In vivo (STZ-induced diabetic rats)	25, 50 or 75 mg/kg for 8 weeks		[104]
Coptisine	Alkaloid	In vivo (STZ-induced diabetic rats)	25 or 50 mg/kg/day for 8 weeks		[105]
L-ergothioneine and metformin	Sulfur Compound Amidine	In vivo (STZ-induced diabetic rats)	35 mg/kg of L-egt, 500 mg/kg of metformin or both for 7 weeks		[95]
Ethyl ferulate	Cinnamate	In vivo (STZ-induced diabetic rats)	50, 75, or 100 mg/kg for 4 weeks		[106]
Isoeucommin A	Polyphenol	In vitro (HRMC and RTECs) In vivo (STZ-induced diabetic rat)	In vitro: 31.25, 61.3, or 125 µm for 2 h In vivo: 2.5, 5, or 10 mg/kg/day for 8 weeks		[107]
Kaempferol	Flavonoid	In vivo (STZ-induced diabetic rats)	200 mg/kg for 8 weeks		[108]
Beta-cryptoxanthin	Tetraterpenoid	In vitro (human podocyte cells) In vivo (db/db mice)	In vitro: 10 µm for 2 h In vivo: 10 mg/kg for 6 weeks		[109]
Simvastatin	Naphthalene	In vivo (STZ-induced diabetic rats)	10 mg/kg for 8 weeks		[110]
Saxagliptin	Peptide	In vivo (STZ-induced diabetic rats)	10 mg/kg 45 min before ischemia		[111]
Maackiain	Isoflavonoid	In vivo (STZ-induced diabetic rats)	10 or 20 mg/kg for7 weeks		[112]
Trametenolic acid	Triterpene	In vivo (db/db mice)	10 mg/kg/day for 4 weeks	Modulate NRF2-mediated downregulation of Nf-kB	[113]
Akebia saponin D	Saponin	In vitro (HK-2 cells) In vivo (STZ-induced diabetic rats)	In vitro: 15, 30 or 60 µg/mL In vivo: 50, 100, or 150 mg/kg for 8 weeks		[114]
Salvianolic acid	Cinnamate	In vivo (STZ-induced diabetic rats)	3 mg/kg for 18 weeks		[96]
Carnosic acid	Diterpene	In vitro (SV40 MES 13 cells) In vivo (STZ-induced diabetic mice)	In vitro: 2.5, 5, 10, or 15 µm for 24 h In vivo: 15 or 30 mg/kg/day for 20 weeks		[115]
Phloretamide	Phenolic amide	In vivo (STZ-induced diabetic rats)	200 mg/kg, twice a week for 12 weeks		[116]
Gentisic acid	Hydroxybenzoate	In vivo (STZ-induced diabetic rats)	100 mg/kg for 2 weeks		[117]
Eriodictyol	Flavonoid	In vivo (STZ-induced diabetic rats)	20 mg/kg for 12 weeks		[118]
Resveratol	Stilbenoid	In vivo (STZ-induced diabetic rat)	5 mg/kg/day for 30 days	Modulate KEAP1	[119]
AB-38b	Aromatic diester	In vitro (mouse GMCs) In vivo (STZ-induced diabetic mice)	In vitro: 2.5, 5, or 10 μm In vivo: 10, 20, or 40 mg/kg/day for 8 weeks		[120]
Epigallocatechin-3-gallate	Flavonoid	In vitro (NRK-52E cells) In vivo (STZ-induced diabetic rats) In vivo (STZ-induced diabetic mice)	In vitro: 5 μm for 24 h In vivo: 100 mg/kg for 30 days In vivo: 100 mg/kg for 24 weeks		[90,91]
Shenkang Injection	Herbal extract	In vivo (STZ-induced diabetic rats)	5 mL/kg for 16 weeks		[121]
Icariin	Flavonoid	In vitro (human glomerular mesangial cells) In vivo (STZ-induced diabetic rats)	In vitro: 1, 3, or 10 μm for 48 h In vivo: 20, 40, or 80 mg/kg for 9 weeks		[122]
Mexacalcitol	Steroid derivative	In vivo (spontaneously diabetic torii rat model)	0.2 μg/kg 3 times/week for 10 weeks		[89]
Chrysophanol	Quinone	In vivo (STZ-induced diabetic mice)	2.5, 5, or 10 mg/kg/day for 8 weeks		[123]
Tilianin	Glycoside	In vivo (STZ-induced diabetic rats)	10 or 20 mg/kg for 28 days		[124]
Caffeoylisocitric acid	Cinnamic acid	In vitro (human GMCs)	10 µm for 24 h		[125]
Deferiprone	Pyridine	In vivo (STZ-induced diabetic rats)	50 mg/kg for 16 weeks	Promote nuclear accumulation/translocation of NRF2	[126]
Liraglutide (GLP-1 receptor agonist)	Peptide	In vitro (human glomerular mesangial cells) In vivo (STZ-induced diabetic rats)	In vitro: 100 or 1000 nm for 48 h In vivo: 200 μg/kg/day, duration unknown		[127]
Tert-butylhydroquinone (tBHQ)	Phenol	In vitro (HK2 cells) In vivo (STZ-induced diabetic rats) In vivo (STZ-induced diabetic rats)	In vitro: 10, 20, 30, or 40 μm for 2 h In vivo: 50 mg/kg every other day for 10 weeks 1% tBHQ for 12 weeks		[128,129]
Biphenyl Diester Derivative-39	Aromatic ester	In vivo (STZ-induced diabetic rats)	15 or 45 mg/kg/day for 8 weeks		[130]
MitoQ	Quinone derivative	In vitro (HK-2 cells) In vivo (db/db mice)	In vitro: concentration unknown, treated for 2 h In vivo: 12 mg/kg twice weekly for 12 weeks		[131]
Chlorogenic acid	Carboxylic Acid	In vitro (HBZY-1 cells) In vivo (STZ-induced diabetic rats)	In vitro: 5, 10, 25, 50, 100, or 200 μm for 24 h In vivo: 10 mg/kg/day for 2 weeks		[132]
SP600125	Anthrapyrazolone	In vitro (mouse GMCs) In vivo (STZ-induced diabetic rats)	In vitro: 10 μm for 48 h In vivo: 5 mg/kg every other day for 24 weeks		[133]
Astaxanthin	Carotenoid	In vivo (STZ-induced diabetic rats)	25 mg/kg/day for 12 weeks		[134]
Myricetin	Flavonoid	In vivo (STZ-induced diabetic mice)	100 mg/kg/day for 6 months		[135]
Obacunone	Triterpene	In vitro (NRK-52E cells)	40 μm for 48 h		[136]
Triptolide	Diterpene	In vitro (MPC-5 cells) In vivo (STZ-induced diabetic mice)	In vitro: 10 μm for 48 h In vivo: 100 μg/kg/day for 12 weeks	Inhibit NLRP3 inflammasome	[137]
Solasonine	Steroidal glycoside	In vitro (MPC-5 cells)	5, 10, or 20 μm for 48 h		[138]
Minocycline	Naphthalene	In vitro (human and mouse podocytes) In vivo (db/db mice and STZ-induced diabetic mice)	In vitro: 10 μm for 24 h In vivo: 5 mg/kg/day for 10 weeks		[139]
AB-38b	Aromatic diester	In vitro (mouse GMCs) In vivo (STZ-induced diabetic mice)	Not specified		[120,140]
WJ-39	Synthetic small molecule	In vitro (rat mesangial cells) In vivo (STZ-induced diabetic mice)	In vitro: 1, 10, or 100 μm for 24, 48, and 72 h. In vivo: 10, 20, or 40 mg/kg for 12 weeks		[141]
Berberine	Alkaloids	In vivo (STZ-induced diabetic hamsters)	100 or 200 mg/kg for 8 weeks		[142]
Icariin	Flavonoid	In vitro (MPC-5 cells) In vivo (STZ-induced diabetic rats)	In vitro: 1, 3, or 10 μm for 48 h In vivo: 20, 40, or 80 mg/kg for 8 weeks		[143]
Quercetin	Flavonoid	In vitro (HK-2 cells) In vivo (STZ-induced diabetic rats) In vitro (HK-2 cells) In vivo (STZ-induced diabetic rats	In vitro: 25 μm for 48 h In vivo: 100 mg/kg for 12 weeks In vitro: 6.25, 12.5, 25, 50, or 100 μm for 48 h In vivo: 25 or 100 mg/kg for 12 weeks	Inhibit ferroptosis	[144,145]
Triptolide	Diterpene	In vitro (human podocyte cells) In vivo (db/db mice)	In vitro: 5 nm for 48 h In vivo: 50 μg/kg/day for 8 weeks		[146]
Paricalcitol	Steroids and derivative	In vitro (HK-2 cells) In vivo (db/db mice)	In vitro: 0.1 μm for 48 h In vivo: 0.1 µg/kg 5 consecutive days/week for 10 weeks		[147]
Carnosine	Peptide	In vitro (HK-2 cells) In vivo (STZ-induced diabetic mice)	Not specified		[148]
Empagliflozin	Glucoside	In vitro (HK-2 cells) In vivo (STZ-induced diabetic mice)	In vitro: 500 nm for 24 h In vivo: 10 mg/kg/day for 8 weeks		[149]
Emodin	Quinone	In vitro (HK-2 cells) In vivo (STZ-induced diabetic rats)	In vitro: 40 μm for 48 h In vivo: 12 weeks		[150]
Crocin	Carotenoid	In vivo (db/db mice)	40 mg/kg for 8 weeks	Modulate PI3K/AKT/NRF2	[151]
Carnosine	Peptide	In vitro (MPC-5 cells)	5, 10, 20, or 30 mm for 48 h		[152]
Beta-sitosterol	Steroids and derivative	In vivo (high fat- and sucrose-induced diabetic rats)	20 or 50 mg/kg/day for 30 days	Modulate TGF-β1/Nrf2/SIRT1/p53 pathway	[153]
Gastrodin	Glucoside	In vivo (STZ-induced diabetic mice)	5, 10 or 20 mg/kg for 6 weeks	Modulate AMPK/NRF2 pathway	[154]
Neferine	Alkaloid	In vivo (STZ-induced diabetic mice)	60, 120 or 240 mg/kg for 12 weeks	Reduce expression of miR-17-5p	[155]
Theaflavin-3,3′-digallate	Flavonoid	In vitro (HepG2 and HK-2 cells) In vivo (STZ-induced diabetic rats)	In vitro: not specified In vivo: 10 or 20 mg/kg/day for 6 weeks	Activate Circ-ITCH and NRF2	[156]
Astragaloside IV	Saponin	In vitro (mouse podocyte cells) In vivo (STZ-induced diabetic mice)	In vitro: 0, 0.3, 1.0, 3.0, 10, 20, 40, 80, or 100 μmol/L for 24 h In vivo: 6 mg/kg/day for 10 weeks	Modulate NRF2-ARE/TFAM pathway	[157]
Asiatic acid	Triterpene	In vitro (HK-2 cells) In vivo (STZ-induced diabetic rats)	In vitro: 10 or 20 μm for 24 h In vivo: 10 or 30 mg/kg/day for 10 weeks	Modulate mitochondrial dynamics via NRF2	[158]
Thymoquinone	Quinone	In vivo (STZ-induced diabetic rats)	10 mg/kg/day for 8 weeks	Modulate NRF2/NOX2 pathway	[159]
1, 2, 3, 4, 6-penta-O-galloyl-beta-D-glucose (PGG)	Tannin	In vitro (MPC-5 cells) In vivo (SFZ-induced diabetic rats)	In vitro: 20, 40, or 80 μm for 24 h In vivo: 5 or 20 mg/kg/day for 8 weeks	Suppress MAPK/NF-KB and ERK/NRF2/HO-1	[160]
Fraxin	Coumarin	In vitro (rat GMCs) In vitro (db/db mice)	In vitro: 0, 20, 40, 60, 80, 160, or 320 μm for 24 h In vivo: 25, 50, or 100 6 days/week for 8 weeks	Modulate Cx43-AKT-NRF2/ARE pathway	[161]
Fucoxanthin	Carotenoid	In vitro (GMCs) In vivo (STZ-induced diabetic rats)	In vitro: 2 μm In vivo: 200 mg/kg/day for 12 weeks	ModulateSIRT1/NRF2/HO-1 pathway	[162]
Isoliquiritigenin	Flavonoid	In vitro (NRK-52E cells) In vivo (STZ-induced diabetic mice)	In vitro: 10 or 20 μm In vivo: 10 or 20 mg/kg every other day for 12 weeks	Activate NRF2 in a SIRT1-dependent manner	[163]
Baicalin	Flavonoid	In vivo (db/db mice)	400 mg/kg/day for 8 weeks	Activate NRF2, inhibit MAPK-mediated inflammatory signaling pathway	[164]
Proanthocyanidins	Flavonoid	In vivo (high fat and sugar induced diabetic mice)	5 mg/kg/day for 4 weeks	Activate p38 MAPK, KEAP1/NRF2	[165]
Oligo-fucoidan	Oligosaccharides	In vitro (NRK-52E cells) In vivo (STZ-induced diabetic mice)	In vitro: 100 μg/mL for 24 h In vivo: 300 mg/kg/day for 8 weeks	Modulate SIRT-1/GLP-1R/NRF2-HO-1 pathway	[166]
Sulforaphane	Sulfur compound	In vivo (STZ-induced diabetic mice) In vitro (mouse podocyte cells) In vivo (STZ-induced diabetic podocyte specific Nrf2 KO mice) In vivo (STZ-induced diabetic mice) In vitro (RMCs) In vivo (STZ-induced diabetic rats)	In vivo: 3 months In vitro: 10 μm for 72 h In vivo: 6.5 or 12.5 mg/kg 3 times/week for 12 weeks 0.5 mg/kg 5 days/week for 4 months In vitro: 5 μm for 24 h In vivo: 5 mg/kg/day for 12 weeks	Activate AMPK-mediated lipid metabolic pathways, activate NRF2 via AMPK/AKT/GSK-3β/Fyn, modulate NRF2/PINK1 pathway	[45,167,168,169]
4-O-methylhonokiol	Lignan	In vivo (STZ-induced diabetic mice)	In vivo: 1 mg/kg 5 days/week for 3 months	Activate AMPK/PGC-1a/CPT1B fatty acid oxidation, NRF2/SOF-2-mediated anti-oxidative stress	[170]
Moringa Isothiocyanate	Isothiocyanate	In vitro (HepG2-C8 and HK-2 cells)	1.25, 2.5, or 5 μm for 24 h	Activate NRF2-ARE signaling, suppress inflammation and TGF-β1 signaling	[171]
Hesperetin	Glycoside	In vivo (STZ-induced diabetic rats)	50 or 150 mg/kg/day for 68 days	Modulate NRF2/ARE/Glyoxalase 1 pathway	[172]
Paeonol	Ketone	In vitro (rat GMCs) In vivo (STZ-induced diabetic mice)	In vitro: 5, 10, or 20 μg/mL for 2 h In vivo: 150 mg/kg 6 times/week for 8 weeks	Modulate SIRT1/NRF2/ARE pathway	[173]
MG132	Peptide	In vivo (STZ-induced diabetic mice) In vitro (HK11 cells) In vivo (OVE26 diabetic mice model)	10 μg/kg/day for 4 months In vitro: 2 μm for 9 h In vivo: 10 μg/kg/day for 3 months	Inhibit proteasomal activity resulting in upregulation of NRF2	[174,175]
Polydatin	Glucoside	In vitro (rat GMCs) In vivo (STZ-induced diabetic mice)	In vitro: 10, 20, or 40 μm for 2 h In vivo: 100 or 200 mg/kg/day for 8 weeks	Modulate CKIP-1/NRF2/ARE pathway	[176]
Sodium butyrate	Fatty acid	In vivo (STZ-induced diabetic mice)	5 g/kg/day for 20 weeks	Inhibit histone deacetylase (HDAC) activity to elevate expression of NRF2, independent of KEAP1 and nuclear translocation	[177]
Sitagliptin	Pyrazine	In vitro (HK-2 cells) In vivo (Goto–Kakizaki rats)	In vitro: 0.05 or 0.1 μm for 1 h In vivo: 10 mg/kg/day for 20 weeks	Downregulate miR-200a/KEAP1/NRF2 pathway	[178]
AICAR	Purine nucleotide	In vitro (MCT cells) In vivo (db/db mice)	In vitro: 2 mm for 48 h In vivo: 2 mg/kg 5 days/week for 4 weeks	Activate AMPK, OGG1 and NRF2 expression	[179]
Gynostemma pentaphyllum saponins	Saponin	In vivo (STZ-induced diabetic rats)	200 or 400 mg/kg/day for 40 days	Activate NRF2 pathway, increased SOD and GSH activity	[180]
Fenofibrate	Aryl carboxylic acid derivative	In vivo (STZ-induced diabetic mice)	100 mg/kg every other day for 3 months	Upregulate FGF21, stimulating P13K/AKT/GSK-3β/Fyn-mediated NRF2 activation	[181]

### 4.2. Alport Syndrome (AS)

AS is a hereditary kidney disease characterized by progressive glomerulosclerosis, inflammation, and fibrosis. The genetic basis of AS is well established, although molecular and cellular mechanisms of disease pathogenesis remain elusive. Direct implication of NRF2 in AS remains underexplored. However, known injury pathways in AS intersect mechanically with NRF2 signaling. These include ROS accumulation, which is a well-documented secondary insult in AS, mitochondrial dysfunction, inflammation [182,183], and most importantly, interstitial fibrosis as one of the final common pathways leading to kidney failure [184,185].

Currently, there are no preventive or curative therapies specifically approved for AS. The standard care remains focused on blocking the Renin–Angiotensin–Aldosterone System (RAAS) to slow the progression of kidney disease. To date, limited studies have explored NRF2 as a therapeutic target in AS; this could possibly be due to the uncertain benefits of NRF2 modulation in glomerular disease [186]. Notably, a study by Kaseda et al. developed UBE-1099, a noncovalent, reversible, and orally available NRF2 activator [187]. Unlike traditional electrophilic agents, UBE-1099 functions by directly inhibiting the KEAP1-NRF2 protein–protein interaction in a noncovalent manner, thereby enhancing the selectivity of the compound while reducing adverse side effects. However, there was a transient increase in proteinuria after the start of administration, similar to studies with bardoxolone methyl, although the study confirms that UBE-1099 did not worsen early renal pathology. Regarding the use of bardoxolone methyl, it was concluded that the CARDINAL phase 3 trial (NCT03019185) did not adequately demonstrate bardoxolone’s effectiveness at slowing kidney disease progression in AS [188,189]. The FDA also raised safety concerns regarding worsening albuminuria and hypertension [190]. There is also lack of data regarding the use of bardoxolone in animal models or other clinical trials of AS [191].

Crucially, genetic overactivation of NRF2 has been found to worsen AS kidney injury in mice, with increased biomarkers of glomerular and tubular injury, inflammation, and interstitial fibrosis [87]. This alludes to the importance of achieving a balanced and controlled activation of NRF2 particularly in AS, where both insufficient and excessive NRF2 signaling has been shown to be maladaptive. Thus, NRF2 modulators with selective and reversible activity may hold therapeutic potential in AS. Despite these insights, NRF2 as a therapeutic target in AS remains underexplored, and further studies are needed to better define the therapeutic window and NRF2 modulation in AS.

### 4.3. Autosomal Dominant Polycystic Kidney Disease (ADPKD)

ADPKD is the most common inherited cause of CKD, characterized by fluid-filled cysts in the kidneys as disease progresses. Studies have found that inflammation plays a role in cyst development and disease progression in early ADPKD. Lu et al. [83] have reported significant reductions in NRF2 in biopsy tissues from ADPKD patients with CKD stages 1 to 3b. Interestingly, this means that NRF2 levels are already reduced in early stages of CKD in ADPKD patients. The abundance of KEAP1 and GSK-3β in mouse kidneys with ADPKD strongly implies that increased degradation of NRF2 protein is the main reason for the repression of NRF2 activity in cystic cells.

There are a limited number of studies that have investigated the role of pharmacological compounds in attenuating cyst formation. Most notably, bardoxolone methyl has been shown to inhibit cyst formation in the MDCK cyst model. Its phase 2 trial (PHOENIX; NCT03366337) has shown efficacy in reducing eGFR, with mechanistic data suggesting reductions in inflammation, ROS, and mitochondrial function in ADPKD [192]. Nevertheless, the planned phase 3 trial (FALCON; NCT03918447) was terminated in 2023 following results from the AYAME phase 3 trial in DKD (NCT03550443) which failed to demonstrate a clear long-term benefit in reducing the occurrence of ESKD. No significant safety concerns were identified. Obacunone, a candidate compound for ADPKD drug development, has been shown to inhibit cyst formation and expansion in various cyst models, including a kidney-specific *Pkd1*-knockout mice model. Its therapeutic effect is mediated through activation of the NRF2 pathway, leading to the suppression of lipid peroxidation via upregulating GPX4 and downregulating mTOR and MAPK pathways [193]. The study by Lu et al. showed that sulforaphane-mediated activation of NRF2 ameliorated cyst growth in mouse models, with treated mice having fewer and smaller cysts, slower kidney enlargement, and improved renal function [83]. Epigallocatechin-3-gallate (EGCG), a natural antioxidant compound produced by plants, has been shown to upregulate *Nrf2* gene expression, increasing the antioxidant capacity of mouse renal tubular epithelial cells [194]. While this study did not directly investigate the effects of EGCG on cyst formation, other research has demonstrated beneficial effects of EGCG in kidney-related conditions such as diabetic nephropathy and lupus nephritis [195]. Given these findings, the effect of EGCG on cyst formation in tubular epithelial cells could be worth further investigation.

To date, there is no conclusive evidence that NRF2 overactivation contributes to cyst formation in ADPKD. Since repression of NRF2 begins in early ADPKD, restoring NRF2 activity in the early disease stage offers a promising therapeutic window in reducing cyst formation and improving renal function, without triggering the adverse consequences associated with prolonged NRF2 overactivation. It is important to note, however, that most pharmacological interventions targeting NRF2 in ADPKD remain in the early stages of investigation and have primarily been evaluated in murine models.

### 4.4. Lupus Nephritis (LN)

Oxidative stress and inflammation are central pathological components of LN, and increasing evidence implicates NRF2 dysregulation as a key contributor to disease progression [196]. Experimental models support NRF2’s protective role in LN: some models show that NRF2-deficient mice develop severe lupus-like nephritis characterized by interstitial inflammation and immune complex deposition [197,198,199], while others show that NRF2 deficiency exacerbates LN [200]. A large study of LN kidney biopsies reported active NRF2 antioxidant responses accompanied by a significant increase in NRF2 and its downstream target NQO1 [85], suggesting that NRF2 is activated in early stages of LN as a compensatory response to oxidative injury. Interestingly, while this study proposed a protective role for NRF2 in disease progression, they found no significant correlation between severity of LN and expression of NRF2. Another study showed that NRF2 levels decreased with worsening renal function (lower eGFR), although the difference was not statistically significant compared to healthy controls [201]. Taken together, these findings indicate that NRF2 is actively upregulated in early LN and may be exhausted in advanced disease rather than actively suppressed.

The therapeutic potential of NRF2 modulators in LN has been explored in experimental models, with a wide range of compounds tested. In LN mice models, sulforaphane reduced the expression levels of inflammatory markers such as TGF-β1 and fibronectin via activation of NRF2 [85]. Administration of EGCG induced NRF2 antioxidant signaling and inhibited NLRP3 inflammasome activation to prevent development of LN in lupus-prone mice [202]. In these mice models, administration of EGCG protected against renal function impairment and renal lesions [195]. Other compounds studied include dimethyl fumarate [203], baicalein [204], extra virgin olive oil [205], and dietary oleuropein [206], all of which act through activating NRF2 pathways to alleviate pristane-induced LN in mice models. Another compound, esculetin [207], works by downregulating the complement cascade and upregulating NRF2, attenuating renal impairment in LN mice. Citral [208] and antroquinonol [209] were tested in accelerated and severe LN mouse models, showing potential benefits for ameliorating late-stage disease. While many NRF2 compounds have been tested in the LN field, testing in clinical settings and in human cell models have been limited.

Overall, since NRF2 activity is upregulated in early LN, therapeutic modulation that augments this endogenous response may potentially be effective in early disease. In late-stage LN, the value of NRF2 activation depends on the cause of low NRF2 levels. If the NRF2 system is exhausted as suggested by Li et al. [201], NRF2 activation in late-stage LN could rescue NRF2 signaling to ameliorate late-stage disease progression, as tested in accelerated and severe LN mouse models [208,209]. However, if the dysregulation is due to irreversible signaling dysfunction, pharmacologic activation alone may be insufficient, necessitating alternative therapeutic strategies.

### 4.5. Adverse Aspects of NRF2 Activation

While restoring normal NRF2 activity is conventionally protective, excessive or untimely NRF2 activation can produce adverse effects. As discussed above, preclinical and clinical studies have revealed potential pitfalls of NRF2 overactivation. One of the clearest adverse outcomes is that strong activation of NRF2 can induce or worsen proteinuria, especially after significant glomerular damage [86]. This has been observed in mice models of proteinuric kidney injury such as Alport syndrome [87,187]. Similarly, in diabetic models, NRF2 overexpression can cause increased albuminuria if the underlying hyperglycemia and glomerular pressures are not controlled [8]. This is especially concerning in the context of kidney diseases, as proteinuria can accelerate kidney injury.

The pro-inflammatory effects of NRF2 can also be context dependent, and overactivation of NRF2 can paradoxically promote inflammation. For example, in a study of atherosclerosis with CKD, persistent NRF2 activation in macrophages triggered the NLRP3 inflammasome, contributing to chronic inflammation [210]. This has also been seen in contexts outside of kidney disease. In systematic lupus erythematosus (SLE) T-cell studies, aberrant activation of NRF2 was reported to suppress regulatory T cell function [211]. These studies underscore the complexity and cell type specificity of NRF2’s role in inflammation.

Additionally, continuous overactivation of NRF2 can result in maladaptive metabolic changes. For instance, a study has shown that high glucose can activate NRF2 in diabetic mice models, which suppresses angiotensin-converting enzyme 2 and boosts angiotensin II. This subsequently contributes to diabetic hypertension and fibrosis [212]. Chronic overactivation of NRF2 in the liver has also been linked to impaired injury repair and insulin resistance [213,214].

Overall, these studies illustrate that NRF2 activation is not universally beneficial both in and outside of the kidney context, and overstimulation can result in adverse effects. These findings argue for a calibrated approach to NRF2 modulation.

## 5. Targeting Fibrosis—The Key Mechanism Underlying CKD Progression

Fibrosis represents a common final pathway across all CKD etiologies. Numerous studies have demonstrated that reduced NRF2 expression is present in the pathogenesis of renal fibrosis and CKD progression. For example, in UUO mice models, renal fibrosis was significantly more severe in *Nrf2*-knockout mice compared to wild-type controls [60]. Similarly, a study by Xu et al. reported decreased NRF2 levels alongside increased expression of fibrosis markers such as collagen and a-SMA in CKD rat models [215]. Furthermore, a recent study also showed that loss of an upstream transcription factor ARNTL resulted in disruption of NRF2 transcription and was associated with fibrosis in CKD patients. In mice models, *Arntl*-knockout mice had severe renal dysfunction and fibrosis. Collectively, these findings imply that reduced NRF2 expression is consistently associated with severity of fibrosis.

NRF2 modulators can target renal fibrosis in CKD through multiple mechanisms. Most commonly, these agents activate the canonical NRF2/HO-1 pathway, thereby mitigating oxidative stress and inflammation. By reducing ROS, they provide indirect protection against fibrotic progression [216]. Beyond its effects on oxidative stress, NRF2 modulators have also been shown to impact other pathways involved in fibrosis. These mechanisms include suppressing epithelial–mesenchymal transition (EMT). For example, a study by Wang et al. showed that NRF2 activation suppressed EMT in renal tubular cells, leading to reduced fibrosis [217]. NRF2 activation was achieved using EGCG, which has also been shown by other studies to be protective against renal fibrosis [218,219]. Other mechanisms involve suppressing the TGF-β1 signaling pathway, as well as through inhibiting profibrotic gene expression. NRF2 modulators targeting renal fibrosis and their mechanisms are summarized in Table 2.

Renal fibrosis is characterized by excessive extracellular matrix deposition and tissue remodeling, making it difficult to reverse once fully established [220]. NRF2 alone is insufficient to reverse existing fibrosis and primarily functions to prevent further oxidative damage and disease progression. Hence, in fibrotic CKD, the optimal therapeutic window for NRF2 modulators would likely be in early fibrosis, prior to extensive inflammation and the accumulation of uremic toxins that may irreversibly impair the NRF2 response.

**Table 2:** NRF2 modulators targeting renal fibrosis and their mechanisms.

Compound	Chemical Class	Study Design	Dosage and Duration of Treatment	Pathway/Mechanism	Reference
Geniposidic acid	Glucoside	In vitro (NRK-52E cells) In vivo (tubulointerstitial nephropathy rat model)	In vitro: 0, 1, 10, 20, 40, 80, 100, or 200 μm for 24 h In vivo: 20 mg/kg/day for 3 weeks	Repressing aryl hydrocarbon receptor (AHR), inhibit NF-kB, activate NRF2	[221]
Betaine	Amine	In vivo (Doxorubicin-induced nephrotoxicity rat model)	200 or 400 mg/kg for 28 days	Downregulate inflammatory and fibrotic gene expression	[222]
Catalpol	Glucoside	In vitro (NRK-52E cells) In vivo (aristolochic acid-induced kidney injury rat model)	In vitro: 5 or 10 μm for 24 h In vivo: 10 or 100 mg/kg/day for 29 days	Activate NRF2, inhibit NF-kB	[223]
Icariin	Flavonoid	In vitro (HK-2 cells) In vivo (UUO mice model)	In vitro: 50 μm for 24 h In vivo: 50 mg/kg/day for 14 days	Activate NRF2/HO-1 pathway, attenuate mitochondrial dysfunction, decrease profibrotic gene expression	[224]
Fasudil	Sulfur compound	In vitro (HK-2 cells) In vivo (hyperuricemic mice model)	In vitro: 50 μm for 48 h In vivo: 5 or 9 mg/kg/day for 5 weeks	Activate NRF2 via NEH2 domain, suppress EMT	[225]
Melatonin and Zileuton	Amine Amide	In vitro (HKC-8 and HK-2 cells) In vivo (UUO mice model)	In vitro: 1 mm melatonin and 5 μm zileuton In vivo: 20 mg/kg/day melatonin, 20 mg/kg/day zileuton or both for 1 week	Upregulate AKT/mTOR/NRF2 pathway	[226]
Spermidine	Amine	In vitro (HK-2 cells) In vivo (UUO mice model)	In vitro: 20 μm for 24 h In vivo: 10 mg/kg/day for 2 weeks	Activate NRF2 and suppress fibrotic signals	[227]
Bixin	Carotenoid	In vivo (carbon tetrachloride-induced renal injury mice model) In vitro (HK-2 cells) In vivo (UUO mice model)	100 or 200 mg/kg/day for 4 weeks In vitro: 0, 20, or 40 μm for 4 or 24 h In vivo: 100 mg/kg once every 3 days for 7 days	Activate NRF2/TLR4/MyD88 pathway, suppress PPAR-gamma/TGF-β1/SMAD3 pathway Suppressing NRF2 ubiquitination, suppress EMT	[228,229]
Saponins from Panax japonicus	Saponin	In vivo (naturally aging rats)	10 or 60 mg/kg/day for 3 or 6 months	Activate NRF2/ARE pathway, suppress NF-kB and TGF-β1/SMAD pathway	[230]
Dihydroquercetin	Flavonoid	In vitro (NRK-49F) In vivo (UUO mice model)	In vitro: 80 μm for 1 h In vivo: single dose of 50, 100, or 200 mg/kg	Activate NRF2 pathway, suppress TGF-β1/SMAD pathway	[231]
Gastrodin	Glucoside	In vivo (carbon tetrachloride-induced renal injury mice model)	200 or 400 mg/kg/day for 4 weeks	Activate AMPK/NRF2/HMGB1 pathway, inhibit NF-kB and TGF-β1 pathways	[232]
Roxadustat	Isoquinoline	In vivo (folic acid-induced kidney injury mice model)	Single dose of 10 mg/kg	Activate AKT/GSK-3β/NRF2 pathway, decreasing ferroptosis	[233]
Bardoxolone methyl	Triterpene	In vitro (mouse GMCs) In vivo (aristolochic acid-induced injury mice model)	In vitro: 0.025, 0.05, or 0.1 μm for 24 h In vivo: 5 or 10 mg/kg/day for 16 days	Activate NRF2/SMAD7 pathway, downregulate TGF-β/SMAD/ECM expression	[63]
Epigallocatechin-3-gallate	Flavonoid	In vitro (MDCK cells) In vitro (NRK-52E cells)	25 μm for 1 h 0, 1, 2, or 5 μm for 48 h	Activate NRF2/HO-1 pathway, protect against EMT and inflammation	[217,218,219]
Carnosic acid	Diterpene	In vitro (NKE cells) In vitro (cadmium-induced nephrotoxic mice model)	In vitro: 5 μm for 24 h In vivo: 10 mg/kg/day for 2 weeks	Activate NRF2/HO-1, Downregulate TGF-β1/SMAD/collagen IV signaling	[234]
Testosterone propionate	Steroids and steroid derivative	In vivo (naturally aging rats)	2 mg/kg/day for 12 weeks	Activate NRF2/ARE signaling, suppress TGF-β1/SMAD signaling	[235]
Oltipraz	Sulfur compound	In vivo (UUO mice model)	30 mg/kg/day for 14 days	Decrease expression of TGF-β1, E-cadherin	[236]
Sinomenine	Alkaloid	In vitro (HEK293 and RAW264.7 cells) In vivo (UUO mice model)	In vitro: 0, 25, 50, or 100 μm for 4 or 24 h In vivo: 100 mg/kg/day for 7 days	Activate NRF2 pathway, inhibit TGF-β/SMAD and WNT/β-catenin pathways	[65]
RTA dh404	Triterpenoid	In vivo (5/6 nephrectomy mice model)	2 mg/kg/day for 12 weeks	Activate NRF2 pathway, suppress NF-kB and TGF-β pathways	[237]
Dimethylfumarate	Carboxylic acid	In vitro (NRK-49F and RMCs) In vivo (UUO mice model)	In vitro: 0, 20, 40, or 80 µmol/l for 1 h In vivo: 25 mg/kg/day for 7 days	Activate NRF2 pathway, inhibit TGF-β/SMAD signaling	[62]

## 6. Current Strategies, Challenges, and Future Perspectives

### 6.1. Current Therapeutic Strategies

Despite the plethora of NRF2 activators that have been tested, only a few have been approved for clinical use in other diseases. These include dimethyl fumarate (Tecfidera), diroximel fumarate (Vumerity), and monomethyl fumarate (Bafiertam), which are electrophilic fumarates that rely on rapid Michael-addition to KEAP1 cysteines to trigger a transient elevation of NRF2 signaling [238,239,240]. These are currently used in the treatment for relapsing multiple sclerosis. Beyond that, Omaveloxolone (Skyclarys) was cleared by the FDA in 2023 as a treatment for Friedreich’s ataxia. Omaveloxolone works by covalently modifying KEAP1 and disrupting KEAP1-CUL3 ubiquitination of NRF2, allowing for reversible elevation of NRF2 [241].

In the context of kidney disease, there is a limited number of ongoing clinical trials for NRF2 modulators targeting kidney disease. CU01-1001 is currently being tested in a phase 2 trial for diabetic nephropathy (NCT05718375) and is a novel pharmacological NRF2 activator that also inhibits TGF-β signaling. Sulforaphane is nutraceutical isothiocyanate that activates NRF2 and is currently being tested in CKD patients in a phase 2 trial (NCT05797506). Notably, other NRF2-targeting compounds like curcumin and resveratrol have previously been tested in phase 2 (NCT03019848) and in phase 3 (NCT02433925) clinical trials, respectively. However, results showed that curcumin was not effective in proteinuria reduction or eGFR preservation in diabetic kidney disease patients [242], and resveratrol did not show antioxidant and anti-inflammatory effects on non-dialyzed CKD patients [243].

### 6.2. Challenges

The development of NRF2-targeted therapies for CKD presents significant challenges ahead. Firstly, the heterogeneity of NRF2 dysregulation across the distinct CKD etiologies results in the variability in therapeutic efficacy. For instance, in AS, genetic overactivation of NRF2 has been shown to exacerbate renal injury, inflammation, and fibrosis. In contrast, no such deleterious effects have been observed in ADPKD, where NRF2 overactivation does not appear to aggravate cytogenesis. Another example of this is seen with bardoxolone methyl: while it has shown no safety concerns in ameliorating cyst formation in ADPKD, it was associated with adverse outcomes in DKD and AS, and remains untested in LN.

Moreover, systemic activation of NRF2 may elicit divergent effects—studies have shown that the NRF2 pathway intersects with a network of 97 cellular pathways [244], forming an intricate regulatory system that may respond differently to different biological contexts. This is exemplified by the escalation of hypertension and hyperglycemia due to NRF2 activation in diabetes, and also in a study by Rush et al. showing increased proteinuria in mice CKD caused by the activation of NRF2 [86].

Compounding these challenges is the limited availability of suitable human models and clinical trials, understandably due to safety and formulation limitations of NRF2-targeting agents. Sulforaphane, one of the most widely studied NRF2 inducers, has been evaluated across various CKD etiologies. However, its clinical translation has been hampered by inconsistencies in formulation, as well as variability in its pharmacokinetics and pharmacodynamics [245]. These issues are not unique to sulforaphane but are broadly representative of the pharmacological challenges facing many investigational NRF2 modulators, including poor solubility and suboptimal delivery systems.

### 6.3. Future Perspectives

To address these barriers, further research could delve into the development of targeted drug delivery platforms that confine NRF2 activation to renal tissues, thereby minimizing systemic off-target effects. It would also be beneficial to investigate the effectiveness and feasibility of combination approaches, which may be useful for late-stage disease. This could also be useful for DKD, where NRF2 modulation alone may exacerbate metabolic dysregulation and lead to adverse side effects. Additionally, a stage-specific modulation strategy may help circumvent the risks associated with indiscriminate NRF2 activation or suppression. Achieving this, however, will require deeper investigation into reliable methods for assessing NRF2 levels and activity across different stages of CKD progression.

## 7. Conclusions

In conclusion, this review presents compelling evidence supporting the therapeutic potential of NRF2-targeted interventions across CKD. It emphasizes that NRF2 activation is not universally effective; rather, its efficacy is highly context-dependent, influenced by factors such as disease etiology, stage, and progression dynamics. These variables must be considered holistically in the development of nuanced and precision-based treatment strategies. Importantly, the therapeutic window for NRF2 modulation appears most advantageous during the early-to-mid stages of CKD and in the initial phase of fibrotic remodeling, where boosting antioxidant defenses may attenuate oxidative damage and favorably alter disease trajectory. Critically, appropriate dosing and vigilant safety monitoring are essential to avoid overactivation and associated adverse effects.

## Abbreviations

The following abbreviations are used in this manuscript:

ADPKD	Autosomal dominant polycystic kidney disease
a-SMA	Alpha-smooth muscle actin
ACACA	Acetyl-CoA carboxylase alpha
ACSL1	Acyl-CoA synthetase long-chain family member 1
AGT	Angiotensionogen
AMPK	AMP-activated protein kinase
AREs	Antioxidant response elements
AS	Alport syndrome
b-TrCP	Beta-transcducin repeat containing E3 ubiquitin protein ligase
CBP/p300	CREB-binding protein
CKD	Chronic kidney disease
COX-2	Cyclooxygenase-2
CRIF1	CR6-interacting factor 1
Cul3-Rbx1	Cullin3-ring box 1
CuZn-SOD	Copper zinc superoxide dismutase
DKD	Diabetic kidney disease
EGCG	Epigallocatechin-3-gallate
eGFR	Estimated glomerular filtration rate
EMT	Epithelial–mesenchymal transition
FASN	Fatty acid synthase
GCS	Glutamylcysteine synthetase
GFR	Glomerular filtration rate
GSH	Gluthathion
GSK-3β	Glycogen synthase kinase-3β
GST	Glutathione S-transferase
HA	Hippuric acid
HO-1	Heme oxygenase-1
IL-1β	Interleukin 1 beta
IL-6	Interleukin 6
KEAP1	Kelch-like ECH-associated protein 1
LN	Lupus nephritis
MAPK	Mitogen-activated protein kinase
MCP-1	Monocyte chemoattractant protein-1
MRPL12	Mitochondrial ribosomal protein L12
NQO1	NADPH-dehydrogenase quinone 1
NRF2	Nuclear factor erythroid 2-related factor 2
PBUT	Protein-bound uremic toxin
PPP	Pentose phosphate pathway
ROS	Reactive oxygen species
SCD1	Stearoyl-CoA desturase 1
SGLT2	Sodium-glucose cotransporter 2
SIAH2	Seven in absentia homolog 2
SLE	Systematic lupus erythematosus
sMAF	Small musculo-aponeutoric fibrosarcoma
TGF-β1	Transforming growth factor beta 1
TNF	Tumor necrosis factor
UUO	Unilateral ureteral obstruction
XRE	Xenobiotic response element
